# Advancements in CO_2_ Capture and Storage: Technologies, Performance, and Strategic Pathways to Net-Zero by 2050

**DOI:** 10.3390/ma19081497

**Published:** 2026-04-08

**Authors:** Ahmed A. Bhran, Abeer M. Shoaib

**Affiliations:** 1 Chemical Engineering Department, College of Engineering, Imam Mohammad Ibn Saud Islamic University (IMSIU), Riyadh 11432, Saudi Arabia; 2 Petroleum Refining and Petrochemical Engineering Department, Faculty of Petroleum and Mining Engineering, Suez University, Suez 43512, Egypt; a.shoaib@suezuni.edu.eg

**Keywords:** carbon capture and storage (CCS), net-zero emissions, Weighted Sum Model (WSM), CO_2_ technologies, multi-criteria decision making

## Abstract

In order to reach net-zero by 2050, we need to have strong decarbonization policies, especially in hard-to-abate clean-ups like steel (8% of the global emissions), cement (7%), and power generation (30%), and negative emissions through direct air capture (DAC) and bioenergy with carbon capture and storage (BECCS). This review paper summarizes the progress in CO_2_ capture, compression, transportation, and storage technologies between 2020 and 2025, including energy penalty (20–40%) and cost (15–30%) reductions, with innovations such as metal–organic frameworks (MOFs), bio-inspired catalysts, ionic liquids, and artificial intelligence (AI)-based optimization. This paper, as a new input into the carbon capture and storage (CCS) field, uses the Weighted Sum Model (WSM) as a multi-criteria decision-making tool to rank the best technologies in the capture, storage, monitoring, and transportation sectors. The weights of the criteria are calculated based on Shannon entropy, and the assessment is performed in three conditions, namely, optimistic, pessimistic, and expected. The weights are computed with sensitivity analysis to make the assessment robust. The viability of key projects, such as Northern Lights (Norway, 1.5 MtCO_2_/year), Porthos (The Netherlands, 2.5 MtCO_2_/year), Quest (Canada, 1 MtCO_2_/year), and Petra Nova (USA, 1.6 MtCO_2_/year), is evident, and it is projected that, globally, CCS will reach 49 MtCO_2_/year across 43 plants in 2025. The review incorporates socio-economic and environmental justice, including barriers such as high costs ($30–600/MtCO_2_), energy penalties (1–10 GJ/tCO_2_), and opposition between people (20–40% in EU/US). In comparison with previous reviews, this article has a more comprehensive focus, provides quantitative synthesis through WSM, and discusses the implications for researchers, policymakers, and stakeholders towards achieving faster CCS implementation on the path to net-zero.

## 1. Introduction

Atmospheric CO_2_ levels increase day by day, reaching 422 ppm in 2025. This consequently requires climate mitigation strategies to keep global warming to 1.5 °C, as mandated by the Paris Agreement [1]. Therefore, it is important to decarbonize high-emission industries such as cement (7% of global emissions), steel (8%), and power generation (30%) using carbon capture and storage (CCS) technology, which attains negative emissions through DAC and BECCS [2,3]. In CCS, CO_2_ is extracted from point sources (like industrial flue gases) or ambient air, and then compressed to supercritical conditions (>73.8 bar, >31.1 °C). After that, it is transported by pipelines or ships and then stored in geological formations (like saline aquifers or depleted reservoirs), or it can be used for other applications such as concrete curing, enhanced oil recovery (EOR), or synthetic fuels [4]. Projects like Northern Lights (Norway, 1.5 MtCO_2_/year), Porthos (The Netherlands, 2.5 MtCO_2_/year), Quest (Canada, 1 MtCO_2_/year), and Petra Nova (USA, 1.6 MtCO_2_/year) have shown commercial and technical viability, and as of 2025, the total capacity of CCS stands at 49 MtCO_2_/year across 43 operational facilities [5].

CCS complements renewable energy, which struggles to address process emissions from industrial sources, and nature-based solutions like afforestation, which are constrained by land availability ($5–50/tCO_2_) and saturation risks after 20–50 years [6,7]. CCS achieves capture efficiencies of 85–99%; however, it faces challenges, including high costs ($30–600/tCO_2_), energy penalties (1–10 GJ/tCO_2_), and public acceptance barriers (20–40% opposition in regions like the EU and US) [8]. Since the 1970s, CCS has evolved from EOR applications (e.g., Val Verde, USA, 1972) to advanced systems like Sleipner (Norway, 1 MtCO_2_/year since 1996) and Climeworks’ Mammoth DAC plant (Iceland, 0.036 MtCO_2_/year, operational since 2024) [9,10]. Innovations in solvents (e.g., water-lean amines, ionic liquids), membranes (e.g., graphene-based), solid sorbents (e.g., MOFs), and AI-driven process optimization have reduced energy demands by 20–40% and costs by 15–30% since 2020; yet scaling to 1000 MtCO_2_/year by 2050 requires technological breakthroughs, robust policies, and socio-economic strategies [11,12].

This review surpasses prior works by:

- Conducting a meta-analysis of more than 100 studies (2020–2025) to benchmark performance and cost reductions;
- Integrating socio-economic metrics (e.g., job creation of 10,000–50,000 per 100 MtCO_2_/year) and environmental justice considerations (e.g., community impact assessments);
- Presenting a multi-criteria decision-making framework for technology selection;
- Providing a comprehensive roadmap for researchers, policymakers, and industry stakeholders to achieve net-zero emissions, addressing technical, economic, and social dimensions of CCS deployment.

As a new point in this review, different available technologies for CO_2_ capture, storage, and monitoring, in addition to transportation methods, are discussed according to the WSM multi-criteria decision-making method. The choice for the best recommended technology and ordering the rest of the choices are analyzed, based on the WSM method, in optimistic, pessimistic, and expected scenarios. WSM is considered as one of the oldest approaches for addressing multi-objective optimization problems [13]. WSM is favored for its simplified computation and elimination of bias. It facilitates the ranking of the outcome of different choices to determine the optimized condition. Furthermore, it can also be utilized to rank the effectiveness of the various available technologies. It operates by giving numerical weights to the various criteria, then determining an overall score for each option by multiplying the normalized value of each criterion by its assigned weight and adding the results together. The weight of each criterion is determined by the Shannon entropy method (ShE), which depends on utilizing the available data and minimizing subjective bias by depending on the variability within the data [14]. It is proven that the ShE method is effective in identifying the optimal scenario compared to other methods like the Analytic Hierarchy Process (AHP) [15]. Finally, sensitivity analysis on criteria weights is carried out to study the effect of criteria weights on determining the best choice and how it affects the preferential order.

Recent reviews on CCS, published between 2023 and 2025, have primarily focused on describing the technological progress of individual capture routes, regional deployment studies, or specific aspects of the CCS value chain. Although these reviews are informative, they often fail to incorporate a holistic, quantitative multi-criteria decision analysis that evaluates and ranks technologies from all segments of the CCS value chain, including capture, compression, transport, storage, and monitoring. In addition, most of the previous reviews have incorporated a qualitative evaluation of criteria, and sensitivity analysis and socio-economic factors, including environmental justice and acceptability, are often not incorporated or are not linked to decision-support tools for the policymakers, researchers, and stakeholders. The gaps are filled in the current review through the performance of the meta-analysis of more than 100 studies (2020–2025), the introduction of the Weighted Sum Model (WSM) along with objective Shannon entropy-based criteria weighting, and the evaluation of the technology rankings using optimistic, pessimistic, and expected scenarios with full sensitivity analysis. This quantitative cross-sector decision-making methodology, along with the inclusion of socio-economic indicators and environmental justice considerations, offers a robust and transparent decision-making process and strategic roadmap for accelerating CCS implementation to meet the global net-zero emissions target of 2050.

## 2. CO_2_ Capture Technologies

Technologies for CO_2_ capture are tailored to the concentration of CO_2_, energy needs, and context of use (industrial versus ambient air, retrofit versus new build). Between 2020 and 2025, advancements in digital technologies, process integration, and materials have significantly improved performance while reducing costs and improving scalability.

The range of reported energy penalty reductions of 20–40% and cost reductions of 15–30%, obtained in the meta-analysis of 50 peer-reviewed studies and pilot/model outputs published in the period from 2020 to 2025, is not the average of the results of individual studies but rather the range of all the studies, with the values depending on the maturity of the technology, the flue gas composition, and the underlying integration assumption [16,17,18,19]. The information provides trade-offs between commercially mature technologies (post-combustion, pre-combustion, oxy-fuels), and emerging systems (direct air capture (DAC), membranes, solid sorbents, cryogenic) to serve strategic directions to reach net-zero emissions by 2050 [19,20].

Post-combustion capture aims at capturing low CO_2_ levels (3–15%), with energy requirements of 2.5–4 GJ/tCO_2_, costs of $30–100/tCO_2_, and capture efficiencies of 85–95%. It is well-suited to retrofitting existing power plants and industrial facilities because of its commercial maturity and wide scalability (10–100 Mt CO_2_/year): Petra Nova (1.6 Mt CO_2_/year at $60/tCO_2_) and Boundary Dam (1 Mt CO_2_/year at $80/tCO_2_) serve as examples [21]. The low land footprint (0.1–0.3 m^2^/tCO_2_) ensures a minimal impact on the environment; however, solvent loss (1–2% loss/year) and amine emissions (0.1–1 ppm) are problematic. Utilization of water-lean amines and bio-inspired catalysts leads to less regeneration energy (20%) and enhanced absorption kinetics (40%), which consequently improve cost-effectiveness to $30–70/tCO_2_ by 2030 [17,22]. Increased acceptance of Virtual Reality (VR)-based public engagement, in pilot areas (70%), would tackle the environmental justice issue of equal siting [23,24].

Pre-combustion capture, which applies to larger CO_2_ concentrations (15–40%), has lower energy usage (1.5–2.5 GJ/tCO_2_), providing recovery rates of up to 98%, but is costly ($40–120/tCO_2_) because of gasification infrastructure. Its scalability (5 to 50 Mt CO_2_/year) and hydrogen co-production (1 t H_2_ per 8 t CO_2_) increase the feasibility of new-build integrated gasification combined cycle (IGCC) plants, as demonstrated in the Kemper County project in the USA, which was designed to capture 3 MtCO_2_ per year at $100/tCO_2_, although it was canceled in 2017 due to cost and technical issues [11,25]. Retrofit applications are constrained by the larger land footprint (0.2–0.5 m^2^/tCO_2_) and high capital costs ($1–2 billion/500 MW plant). The calcium looping and high-temperature sorbents enhance the cycle stability by 25–30 years, decreasing the cost by 25% [26,27]. Community benefit arrangements resolve 30–40% of local issues, and they support rescannable deployment [28].

Oxy-fuel combustion is most effective in high CO_2_ concentrations (80–90%), at 95–99% efficiency with an energy requirement of 1.0–2.0 GJ/tCO_2_ (including air separation unit (ASU) cost) and a cost of $35–110/tCO_2_. The Callide project (Australia, 0.1 Mt CO_2_/year for $70/tCO_2_) and the Schwarze Pumpe (Germany, 0.24 MtCO_2_/year at an efficiency of 99%) [29,30] demonstrate its commercial maturity and scalability (5–50 Mt CO_2_/year). Energy reduction of ASU by chemical looping combustion (CLC) and ion transport membranes (8–12%) and the efficiency enhancement of waste heat recovery (8–12%) [31,32,33] are considered advantageous for using oxy-fuel combustion technology. However, higher land footprint (0.3–0.6 m^2^/tCO_2_), and retrofitting costs (500–800/kW) are obstacles, but they could be overcome by enhancing public acceptance with VR engagement up to 70–80% [34,35].

Direct air capture (DAC) reaches ultra-dilute CO_2_ (0.04%), including high energy requirements (4.0–10.0 GJ/tCO_2_) and costs ($100–250/tCO_2_) at 85–90% efficiency. It is limited to specific scalability (0.1–10 MtCO_2_/year) at the demonstration stage, and the large land footprint (0.5–1.0 m^2^/tCO_2_) needs to be carefully sited. DAC, however, has actual negative emission potential (−0.5 to −1 tCO_2_/tCO_2_ when powered by low-carbon energy sources), unlike bioenergy with carbon capture and storage (BECCS), which is a different method of net negative emissions. This is essential to achieve net-zero targets [19,36,37,38,39,40,41,42]. MOFs and renewable integration cost less than Climews Mammoth (Iceland, 0.036 MtCO_2_/year at 150/tCO_2_) and 1PointFive (USA, 0.5 MtCO_2_/year at 150/tCO_2_) [19,36,37,38,39,40,41,42]. AI optimization reduces energy by 15–20% with a goal of 80–100/tCO_2_ by 2030 [36,43,44,45]. Community-based site selection improves environmental justice, and 30–40% of the community issues are considered [34].

Membrane-based and solid sorbent technologies have low energy (0.5–2.0 GJ/tCO_2_ in membranes; 1.5–2.0 GJ/tCO_2_ in sorbents). Solid sorbents achieve 90–95% efficiency at a cost of $40–80/tCO_2_ for solid sorbent compared to 85–90% efficiency at cost of $50–80/tCO_2_ for the membrane-based method. Their maturity and scalability (1–20 MtCO_2_/year) are limited by small-scale industrial applications (<1 MtCO_2_/year). The low land requirements (0.05–0.15 m^2^/tCO_2_) enable the use of space-constrained industrial applications (including those operating at scales below 1 MtCO_2_/year), as demonstrated by the air liquid Membrane Technology and Research (MTR) Polaris (USA, 0.1 MtCO_2_/year at $60/tCO_2_) and National Energy Technology Laboratory (NETL) sorbent (USA, 0.05 MtCO_2_/year at $50/tCO_2_) projects [46,47,48,49,50]. Graphene membranes and AI-designed MOFs increase selectivity (CO_2_/N_2_ > 100) and capacity (3–4 mmol/g), but dilute streams (<5% CO_2_) are difficult to achieve [51,52].

Cryogenic capture is applicable at 15–20% CO_2_ with moderate energy (2.03 GJ/tCO_2_) and costs ($50–100/tCO_2_) with 90–95% efficiencies. It can operate at demonstration level with a scalability of 1–10 MtCO_2_/year. Its land footprint (0.2–0.4 m^2^/tCO_2_) is moderate because it only requires conventional industrial plot sizes for liquefaction equipment and storage tanks (smaller than DAC, larger than membrane systems) [53,54,55,56,57,58,59,60], and hybrid systems with waste heat recovery cut costs to $40–70/tCO_2_, scaled to 0.01 MtCO_2_/year by CryoCap, run by Sustainable Energy Solutions (USA, 0.01 MtCO_2_/year at $60/tCO_2_) [53,54,55,56,57,58,59,60]. Dilute Stream Energy intensity restricts wider application, whereas its applicability to industrial applications (e.g., liquified natural gas (LNG)) justifies consideration of selected applications [61,62,63].

As a summary, Table 1 benchmarks CO_2_ capture technologies innovated or improved between 2020 and 2025, and includes the suitability of CO_2_ concentration, energy use, affordability, capture efficiency, scalability, land area, and technological maturity. The analysis reveals that a wide range of commercial technologies (post-, pre-, and oxy-fuel combustion) dominates near-term scalability and cost-effectiveness. On the other hand, emerging technologies (DAC, membranes, sorbents, cryogenic) provide energy efficiency and negative emissions potential. Material innovations and AI optimization have led to a 25% cost reduction and 30% efficiency gain since 2020, indicating progress toward economic viability [16,17]. Overall, the most common are the post-combustion and oxy-fuel methods because they are easily integrated into existing systems and are already commercially viable. However, the membranes/sorbents and DAC methods are more energy-efficient and provide an opportunity for net-negative emissions, but they are not so easily scaled up. This type of trade-off analysis has shown that hybrid systems are required to meet the cost, energy, and net-negative emissions goals set for 2050.

The evaluation of CO_2_ capture technologies requires a keen consideration of the inherent trade-offs, in which performance measures indicators, such as cost, energy use, capacity, extension, and ecological footprint, should be weighed against the application specificity of the use. Based on a meta-analysis of developments in the 2020–2025 period, the discussion below clarifies trade-offs of major CO_2_ capture technologies, pointing to how innovations overcome constraints and emphasizing existing obstacles to large-scale implementation. This framework guides the best choices based on retrofit, new-build, negative emissions, and low-energy settings and eventually informs strategic paths to net-zero emissions by 2050. The trade-offs and optimal choice of CO_2_ capture technologies have been summarized in the following subsection.

### 2.1. CO_2_ Capture Technologies Trade-Offs and Optimum Selection

With respect to the trade-offs of CO_2_ capture technologies, the post-combustion technology is the least expensive and has high retrofit suitability (e.g., Petra Nova retrofitted a 240 MW plant at $1 billion), but with moderate energy penalties and solvent degradation [21,22]. The pre-combustion method offers low energy and co-produces hydrogen but has high capital costs and is not as well-suited to retrofits [11,25]. Concerning oxy-fuel, it has the highest efficiency and CO_2_ purity (>98%), but faces ASU energy requirements and retrofitting complexity [29,32]. DAC is utilized essentially with negative emissions, but with the highest costs and energy requirements, making it limited to 0.1–10 MtCO_2_/year scalability. The membranes/sorbents have the lowest energy and land area, but do not have industrial-scale use and do not work well with low CO_2_ concentrations [12,17,64,65,66]. Nevertheless, cryogenic technology is highly efficient on a given stream (e.g., LNG) and energy-intensive on dilute gases, with a medium level of scalability [61,62,63].

The optimum technology for CO_2_ capture can be selected according to retrofits, new-builds, negative emissions, and low-energy applications. Post-combustion technology is optimum, based on retrofits, with low retrofit costs (200–400/kW), high scalability, and commercial maturity (e.g., Petra Nova, Boundary Dam). It is countered by water-lean amines and AI optimization with a moderate energy penalty that reaches $30–70/tCO_2_ by 2030 [19,21,22]. Oxy-fuel combustion is most effective in new construction due to its high efficiency, moderate costs, and applicability to coal/biomass plants. The use of CLC and ion transport membranes reduces ASU expenses, as demonstrated in Callide [30,31,32]. DAC using renewables is the only feasible alternative based on the negative emissions. MOFs and renewable integration would push the costs to $100/tCO_2_ with pilots such as 1PointFive demonstrating scalability to 0.5 MtCO_2_/year [36,37,44]. Nonetheless, low energy and compact footprint for membranes and solid sorbents are favored in low-energy applications where space is limited (high CO-density streams, such as cement plants). Pilots such as MTR Polaris and the NETL sorbent project validate $40–80/tCO_2_ [46,47,48,49,50].

### 2.2. Mechanistic Drivers and Structure–Property Relationships in Enhanced CO_2_ Capture

The influence of material structure on carbon capture is crucial, since the physicochemical characteristics of the capture medium govern the energy efficiency as well as the cost of the entire system [67]. While the evolution of carbon capture is often measured by macroeconomic indicators like cost per/ton, it is crucial to take the chemical and physical characteristics of the capture medium into consideration, since it influences the energy efficiency and could also affect the cost of the whole system [68]. The major drivers of these enhancements reside in the particular molecular engineering of capture media [69].

The transition from traditional aqueous alkanolamines to enhanced materials is driven by molecular-level engineering that optimizes the trade-off between CO_2_ capacity and regeneration energy. In metal–organic frameworks features, crystalline structures are designed with large surface areas and open metal sites that act as strong Lewis acid centers; such sites form coordinated bonds with CO_2_ remarkably, improving adsorption enthalpy and selectivity via molecular sieving [70,71]. Similarly, ionic liquids surpass conventional solvents by removing the high latent heat needs of water. They use powerful coulombic interactions from high-charge density cation–anion pairs to polarize CO_2_, while task-specific amine functionalization permits a 1:1 molar capture ratio—doubling the theoretical capacity compared to the 0.5:1 ratio of standard monoethanolamine (MEA) [72]. Additionally, the combination of bio-inspired catalysts, such as Carbonic Anhydrase (CA), marks the kinetic restrictions of energy-efficient but naturally slow solvents as potassium carbonate [73]. The zinc-hydroxide active site in the CA enzyme reduces the energy of activation for CO_2_ hydration to bicarbonate, promoting a decline in regeneration temperatures from ~120 °C to less than 80 °C [74]. By linking physical architectures—including pore size, metal coordination, and chemical tethering, with critical performance metrics like selectivity and parasitic energy load, these innovations establish a mechanistic pathway to overcome the conventional energetic barriers of carbon capture.

### 2.3. Ordering CO_2_ Capture Technologies Based on WSM Multi-Criteria Decision-Making Method

Three scenarios have been examined in this work to select the optimum technology and ordering the rest of technologies. The assumed three scenarios are as follows [13,14,15]:

- **Optimistic:** This scenario depends on examining the available alternatives of carbon capture technologies under the best conditions. In this case, the best conditions assume the existence of the minimum CO_2_ concentration, achieving the minimum capture cost and energy consumption/tCO_2_, minimum land footprint, and maximum efficiency and scalability. The criteria that are not quantified in this case, such as retrofit suitability and negative emissions, have been identified by numbers, ranging from 1 to 5, for low to high possibilities.
- **Pessimistic:** This assumption relies on examining the available alternatives of carbon capture technologies under the worst conditions. In this case, the worst conditions assume the existence of the maximum CO_2_ concentration captured at the maximum cost and energy consumption/tCO_2_, maximum land footprint, and minimum efficiency and scalability.
- **Expected:** In this case, the different capture technologies are assumed to work at middle conditions, which are assumed to be midpoints between the optimistic and pessimistic scenarios.

Table 2 summarizes the various assumptions for each scenario, in addition to the order of preference of each technology using the WSM and ShE multi-criteria decision-making method. The weight of each criterion based on the ShE method is shown in the last row in Table 2. It also clarifies the order of preferences for each capture technology in each scenario. It is worth noting that post-combustion is the optimal technology in all scenarios, which matches with the analysis given in Section 2.4, as it is retrofit-able and scalable.

Although the WSM introduces a robust quantitative ranking based on techno-economic performance, the final assessment incorporates public acceptance and environmental justice as crucial qualitative benchmarks. This social feasibility ensures that the highest-superior pathways are also socially viable for long-term deployment.

### 2.4. Sensitivity Analysis

In this section, the impact of criteria weights on the decision for the optimal solution and order of preferences is introduced. Sensitivity analysis is carried out on the expected scenario. In this regard, 10 scenarios are considered. In the first scenario, the weights of all criteria are considered equally, i.e., 0.125. In the next eight scenarios, one criterion is considered more important than the others. The weight of the criterion with higher importance is considered to be 0.3; this weight (0.3) represents a dominant priority scenario, in which corresponds to more than twice the weight of each of the rest baseline criteria (0.1). This assumption ensures a rigorous test of the model’s stability. Weights of each criterion in the first nine scenarios are presented in Table 3. The last scenario is based on using the weight obtained using the Shannon Entropy method. The weight of each criterion will play an important role in determining the best choice and will affect the preferential order. Table 4 presents the rank of each technology for different scenarios.

It is obvious from the results shown in Table 4 that the optimum solution based on WSM is not sensitive to the criteria weight.

Table 5 gives a merged view of how different CO_2_ capture technologies rank through different decision-making scenarios, from the initial performance assessments to the robust sensitivity analysis.

It is worth noting that post-combustion is the best choice in all scenarios, regardless of whether we are dealing with the optimistic or pessimistic scenario. Moreover, it is chosen as the first choice even if the weights are shifted in sensitivity analysis. This could be attributed to having the best balance of commercial maturity, high scalability (10–100 MtCO_2_/year), and low retrofit costs ($30–100/tCO_2_). On the other hand, ranking the rest technologies differs when using different weights for each criterion. It is also noted that solid sorbents frequently rank second, as shown from the sensitivity analysis; this could be due to their superior energy efficiency ($1.5–2.0~GJ/tCO_2_) and minimal land footprint ($0.05–0.15~m^2^/tCO_2_), making them ideal for space-constrained industrial sites. Direct air capture (DAC), while critical for negative emissions targets, orders lower due to its high current costs ($100–250/tCO_2_) and extreme energy intensity ($4.0–10.0~GJ/tCO_2_). Many high-performing emerging technologies (membranes, sorbents) have recently been limited to demonstration scales ($<1~MtCO_2_/year) and require more industrial validation to compete with commercial solvent-based systems.

Future directions must focus on hybrid systems, quantum computing to discover materials, and policy incentives to close disparities in DAC scalability and deployment in developing nations, with equitable CCS implementation to achieve 1000 MtCO_2_/year by 2050 [40,75].

## 3. CO_2_ Compression and Transportation

Transportation and compression play important roles in delivering CO_2_ to storage facilities, where compression uses 10–15% of CCS energy, and transportation takes 10–20% of the cost. The innovations of 2020–2025 have enhanced efficiency, safety and scalability, and digital technologies have a major part to play [76,77,78].

### 3.1. Compression

Multi-stage centrifugal or integrally geared compressors can be applied to compress CO_2_ to supercritical conditions (>73.8 bar, >31.1 °C) and require 0.3–0.5 GJ/tCO_2_ for $3–8/tCO_2_ [79]. Key advancements include:

- AI-driven dehydration: Removing water (<50 ppm) to protect pipelines from corrosion, saving 5–10% of energy and prolonging pipeline longevity by 10–15 years [80,81,82,83].
- Waste heat recovery: The compressor heat is recycled for solvent regeneration or power generation to enhance efficiency by 8–19% [84,85].
- Integrally geared compressors: Optimization of staging and impeller design leads to decreasing energy by 10–15% (0.25–0.4 GJ/tCO_2_) and maintenance costs by 5–10% [80,85].
- Pilot results: The Quest project (Canada, 1 MtCO_2_/year) compressed CO_2_ at $5/tCO_2_ at 99.9% uptime with AI to optimize pressure cycles [86,87].
- Safety and emissions: The advanced seals can reduce leakage of CO_2_ by 36–54%, which satisfies community safety considerations [79,88]. Compression energy is a major cost driver, and innovations have decreased its percentage from 15% to 10% of the CCS chain since 2020 [4,78]. Environmental justice includes reducing noise and emissions of compressor stations on residential premises [75,89].

### 3.2. Transportation

CO_2_ is either pumped through pipelines (onshore/offshore) or with ships, although pipelines are more prevalent because of their high capacity and low cost. While ships cost $10–20/tCO_2_ for 1000–2000 km, the US transports 70 MtCO_2_ annually over 8000 km at a rate of $2–10/tCO_2_ per 100 km [90,91,92]. Among the advancements are:

- High-strength alloys: using X70/X80 steel improves the pipeline lifetime and decreases maintenance costs; they are considered to be economical methods of transportation [93,94].
- Corrosion management: efficient corrosion inhibition processes like epoxy coating, cathodic protection or coating with environmentally friendly materials could help in mitigating corrosion and preserving the mechanical properties of carbon steel, copper, and aluminum. The inhibition efficiency could reach up to 99.99% [95,96,97].
- Ship design: “dual-purpose” ships carrying LNG-gas in one direction and CO_2_ for storage on the return journey are also under consideration. Thus, for carbon capture utilization and storage (CCUS)-EOR systems, both voyages would be profitable, and energy efficiency would be improved [98]. Moreover, different LNG-like CO_2_ carriers (e.g., 7500 m^3^ capacity (low pressure) or 7500 m^3^ capacity (high pressure)) have significant effects on offshore hub costs, energy consumption, and environmental performance, as proven in the Northern Lights projects (1.5–5 MtCO_2_/year) [99].
- Blockchain tracking: provides an accountable use of CO_2_, raises investor trust and carbon credit markets, and improves tracking, verification, and transparency of carbon credits [100].
- Pilot results: spatial analysis of 35 proposed CCS projects in the US power sector shows 94.3% co-located within 3 miles of environmental justice (EJ) communities, with 85.1% of 497 affected EJ census block groups already under heightened environmental stress from pollutants and hazardous sites. To mitigate CCS supply chain risks—like CO_2_ pipeline leaks burdening low-income communities and communities of color—future projects require EJ safeguards: avoiding vulnerable routes, conducting cumulative impact assessments, ensuring pre-permitting regulatory compliance, implementing fence line monitoring, and enabling community engagement with accessible information, technical support, and veto rights [101].

### 3.3. Comparative Analysis and Optimum Selection

For transportation methods trade-offs, pipelines have the lowest cost ($2–10/tCO_2_ per 100 km), highest capacity (up to 50 MtCO_2_/year), but high land use (10–20 m^2^/km) and public opposition (30–40% in certain areas) due to perceived leak risk (<0.01%/year) [102,103]. Conversely, ships are more expensive ($10–20/tCO_2_) and have less capacity (0.5–10 MtCO_2_/year), but can be offshore-stored, and they have lower land footprint and emissions [104].

In terms of the optimum selection, in the case of onshore/long-distance, pipelines are the best choice, as their costs are low ($2–10/tCO_2_ per 100 km), capacity is high (1–50 MtCO_2_/year), and they are commercially mature (e.g., US Cortez pipeline, 20 MtCO_2_/year). The reliability is guaranteed by digital twins and corrosion management [102,105]. Nonetheless, in terms of offshore/remote areas, ships are favored due to their flexibility and minimal environmental impact, which are suitable in offshore centers such as the Northern Lights (1.5–5 MtCO_2_/year) and areas without pipeline systems (e.g., Southeast Asia) [105,106].

### 3.4. Ordering CO_2_ Transportation Methods Based on the WSM Method

As mentioned earlier, three scenarios are examined. In this section, the optimistic scenario considers the maximum CO_2_ capacity/year, maximum distance, minimum environmental impact, and minimum incidents/year. Table 6 summarizes the various assumptions for each scenario, in addition to the order of preference of each transportation method using the WSM and ShE multi-criteria decision-making method. The weight of each criterion based on the ShE method is shown in the last row in Table 6. It also clarifies the order of preferences for each transportation method in each scenario. MCDM results differ from the results discussed in Section 3.3, in which ships, as a transportation method, are preferred to pipelines. This could be attributed to the higher weights for distance, environmental impacts, and probability of incidents per year, when compared to other criteria, as shown in Table 6.

### 3.5. Sensitivity Analysis

Sensitivity analysis is carried out on the expected scenario in this section by assuming seven scenarios. In the first scenario, the weights of all criteria are considered equally, i.e., 0.2. In the next five scenarios, one criterion is considered more important compared to other criteria. The weight of the criterion with higher importance is considered 0.4, and the weights of the other criteria are 0.15. The weights of each criterion in the first nine scenarios are presented in Table 7. The last scenario is based on using the weight obtained using the Shannon Entropy method. Table 8 presents the ranking of each transportation method for different scenarios.

A consolidated view of the evaluated rank of transportation methods across the different decision making scenarios is presented in Table 9.

It is observed in Table 8 that the decision for the transportation method is sensitive to the weight of the criteria. However, Table 9 presents that most of the results recommend transportation via ship; this result does not match with conventional thinking, in which pipelines are usually considered as the standard for high-capacity onshore transport. However, the proposed WSM analysis, which incorporates environmental impact, safety incidents, and flexibility, recommends ships in most of the proposed scenarios. Ships offer high flexibility for offshore storage hubs (e.g., Northern Lights) and need notably smaller land footprint (10–20~m^2^/km for pipelines versus minimal for shipping lanes). However, ships are still more costly ($10–20/tCO_2_) than pipelines ($2–10/tCO_2_) and have smaller instantaneous capacity (0.5–10 $MtCO_2_/year), making them less suitable for massive, localized industrial clusters. Additionally, the liquefaction needed for ship transport rises the overall energy penalty of the CCS chain.

## 4. CO_2_ Storage Technologies

The long-term sequestration of CO_2_ is guaranteed by geological storage; saline aquifers, depleted reservoirs, and mineral carbonation are ranked according to capacity and permanence. A summary of performance metrics is presented in Table 10. More details and comparative analysis will be discussed in the following subsections.

### 4.1. Saline Aquifers

Deep saline aquifers of more than 800 m capacity provide a large storage capacity of 1000 to 10,000 GtCO_2_ with a storage duration exceeding 99 years, allowing for retention of over 1000 years, making them the main choice for carbon sequestration [107,120]. Commercial projects, like Sleipner in Norway, injecting 1 MtCO_2_ annually since 1996, and Gorgon in Australia, which handles 3–4 MtCO_2_ annually, prove the commercial viability of saline aquifers. Significant progress has been made, including reservoir modeling with machine learning to improve injectivity by 20–25%, optimizing well placement, and obtaining injection rates of 0.5–2 MtCO_2_/per well/year. Offshore hubs with 100–200 MtCO_2_/per year were planned in initiatives such as Northern Lights and Porthos, where shared infrastructure can cut costs by 15–20% [108,109]. Cap rock integrity enhanced through innovative sealing technologies, including geochemical barriers, can reduce leakage to less than 0.001% per year, confirmed by 25 years of Sleipner data [121,122,123]. The integration of biochar, with pilot projects, shows improvements in both storage stability and pressure accumulation reduction [124]. The costs of pilot results of the Illinois Basin-Decatur Project in the USA, with 1 MtCO_2_/year injection, are $57 million for monitoring and storage [125]. This achieves costs of up to 18/tCO_2_, with offshore storage at the higher end due to the drilling cost of $5–10 million/well [126]. In the meantime, the induced seismicity fears expressed by the community (e.g., a risk of events bigger than magnitude 3) are compensated through the real-time seismic monitoring and community participation, leading to 60–80% acceptance rates [107,127].

### 4.2. Depleted Reservoirs

Depleted oil and gas reservoirs leverage existing wells and infrastructure to reduce costs to $4–15/tCO_2_ when combined with enhanced oil recovery (EOR), offering a capacity of 100–1000 GtCO_2_ with leakage rates below 0.001% per year [108,128,129]. Key developments include AI optimization, which enhances injection efficiency by 15–20% and achieves rates of 0.3–1.5 MtCO_2_ per year per well, as demonstrated in the Weyburn-Midale project in Canada injecting 2 MtCO_2_ annually [110,113]. Improvements also consider EOR synergies, which emphasize sustainability and cost reduction. CO_2_-EOR enhances recovery of oil and storage, with injection rates of 1–2 MtCO_2_ per year in mature oil fields, as observed in Canadian projects [130]. Costs can be offset by synergies with EOR, amounting to 0.1–0.5 extra barrels of oil per tCO_2_ injected, which could result in revenues of $20–60/tCO_2_, and lower the total CO_2_ footprint of oil production [131,132]. Techno-economic models show costs of capture of between $60 and $110/tCO_2_, reduced to half in 2030 with post-combustion and pre-combustion routes, and storage costs as low as $8–15/tCO_2_ integrated with EOR [132,133]. The reuse of infrastructure reduces capital costs by 20–40% through current wells and pipelines. Positive environmental effects include 99%+ retention rates over decades, which would require life-cycle evaluation to deal with upstream emissions (0.1–0.4 tCO_2_/barrel) to achieve net reductions. Engagement of communities reduces 30–50% of local issues by creating jobs in energy transition areas [110,130,132].

### 4.3. Coal Seams

With the high adsorption capacity of coal, coal seams have also received a new focus involving CO_2_-enhanced coalbed methane (CO_2_-ECBM), as coal could be used to recover and store methane simultaneously. Recent laboratory studies indicate that CO_2_ injection at pressures of 8–18 MPa can be successfully used to replace methane, but coal-matrix swelling can lower the permeability and decrease injectivity in the long run [134,135]. Examination of ECBM processes highlights competitive adsorption, diffusion driven by pressure, and coal rank effects on storage behavior [136]. Geological tests also show that deep inaccessible seams can store tens of cubic meters of CO_2_ per ton of coal, and basin-scale storage has been reported in areas like Kazakhstan. However, it can only be utilized to a maximum of less than 100 GtCO_2_ at costs varying from $8–25/tCO_2_ and injection rates of 0.01–0.1 MtCO_2_/year per well [124]. The USA Allison Unit pilot at a rate of 0.01 MtCO_2_/year achieves $15/tCO_2_ with 99% retention and recovery of 0.5–1 m^3^ of methane for each injected ton of CO_2_ [137,138].

However, in spite of this possibility, recent techno-economic analysis emphasizes injectivity loss and operational costs as the main challenges [139]. Social-acceptance research also indicates that open communication and early consultation are important factors that enhance the social acceptance of CO_2_ storage projects [34].

### 4.4. Ocean Storage

At depths of more than 1000 m, ocean storage has a huge capacity for sequestering carbon, surpassing 100,000 GtCO_2_, with 95–99% permanence over 100 years, with costs varying from $10 to $30 per ton of CO_2_ [140,141]. However, environmental problems, including ocean acidification, which lowers pH by 0.01 to 0.1, and legal barriers under frameworks like UNCLOS and the London Protocol—which explicitly prohibits the dumping of CO_2_ into the ocean (water column) except for limited scientific research purposes—severely restrict its scalability to 1–50 MtCO_2_ annually [142,143,144]. Pilot tests show no biological impact, with pH fluctuations below 0.1 at depths of more than 1500 m. One example of this is Japan’s Tomakomai project, which injects 0.3 MtCO_2_ annually [143,144]. Monitoring improvements, such as autonomous underwater vehicles that can identify leaks with 95% accuracy, have lowered costs by 10% to 15%, yet public opposition, which ranges from 50% to 70%, highlights the necessity of transparent impact assessments to alleviate worries and improve deployment viability [142,143,144,145,146].

### 4.5. Mineral Carbonation

A highly permanent carbon sequestration option with >99.9% stability and a worldwide capacity of 100–1000 GtCO_2_ is provided by mineral carbonation, which transforms CO_2_ into stable carbonates by reacting it with silicate minerals such as olivine and basalt [147]. The Wallula Basalt Pilot in the USA achieves 95% carbonation in two years at $50/tCO_2_, while the CarbFix project in Iceland demonstrates its potential with an annual capture of 0.1 MtCO_2_ [148,149]. Costs are reduced to $30–60/tCO_2_ by using plentiful industrial wastes such as fly ash and steel slag (1 Gt/year worldwide), and enzymatic or metal-based catalysts speed up reaction kinetics by 25–30%, reducing energy needs by 20% and allowing for the storage of 0.01–0.1 MtCO_2_/year per site [149,150,151]. Co-locating with industrial sites reduces land use disputes and promotes community benefits, while integrating biochar further increases efficiency by 5–10% [152,153]. Despite these developments, scaling is limited to 0.1–10 MtCO_2_/year because of expensive costs and slow reaction kinetics (1–5 years for full carbonation), yet its unparalleled permanence makes it a potential long-term strategy [150,152].

### 4.6. Comparative Analysis and Optimum Selection

Carbon storage alternatives offer diverse trade-offs in terms of capacity, cost and environmental impact, and all these have their settings. Saline aquifers are the most storage-capable (1000–10,000 GtCO_2_) and least expensive ($4–18/tCO_2_) based on geographic location, with 70% storage capacity in North America and Europe, and can be dangerous sources of seismicity (0.1–1% probability of M > 3) [107,127]. Existing infrastructure can be used to store oil and gas at a low cost with increased oil recovery (EOR), generating $10–50/tCO_2_ in revenue, but with reduced capacity (100–1000 GtCO_2_), and the EOR process generates emissions of 0.1–0.3 tCO_2_/barrel [108,130,132]. Coal seams can recover methane (0.5–1 m^3^/tCO_2_) but have reduced capacity (less than 100 GtCO_2_) and increased cost ($8–25/tCO_2_), scaling poorly [137,148]. Ocean storage offers a very large storage capacity (>100,000 GtCO_2_), but is highly hazardous to the environment, in which the pH decreases by 0.01–0.1, and is prohibited by its regulations to make up more than 1% of the total storage capacity in any location [119,140]. Mineral carbonation is both permanently superior (>99.9% permanent) and less damaging to the environment, although costs are high ($30–100/tCO_2_) and reaction kinetics are slow (1–5 years), which limits its scalability [148,150,152,154].

The optimum approaches to carbon storage are based on the objectives of the project, balancing capacity, cost and permanence. Saline aquifers are favored when large storage is required because of its large capacity, low costs, and commercial maturity, as demonstrated by Sleipner and Gorgon; offshore hubs and AI-based modeling make them scalable to 10–200 MtCO_2_/year [107,108,113,124]. Depleted oil and gas reservoirs are best when including enhanced oil recovery (EOR) [110,128,130]. To achieve permanent sequestration, mineral carbonation is the most permanent (>99.9%), especially with the use of industry waste, as shown with CarbFix and Wallula, though it has a cost range that varies between $30 and 60/tCO_2_ [141,147,149].

### 4.7. Ranking CO_2_ Storage Technologies by the WSM Method

The optimistic scenario in this case dealt with the minimum cost and maximum CO_2_ storage capacity, permanence, injection rate, and scalability. Table 11 summarizes the various assumptions for each scenario, in addition to the order of preference of each storage technology in each proposed scenario.

### 4.8. Sensitivity Analysis

In this section, eight scenarios are assumed. In the first scenario, in which the weights of all criteria are considered equally, the weight of each criterion is assumed to be 0.167. In the next six scenarios, the weight of the criterion with higher importance is considered to be0.3, and the weights of the other criteria are 0.14. Weights of each criterion in the first seven scenarios are presented in Table 12. Table 13 presents the rank of each storage technology for different scenarios.

Table 14 provides a summary of the WSM ranking outcomes for storage technologies according to the different resulting scenarios. This summary illustrates the stability of the model through optimistic, pessimistic, and expected scenarios, as well as the average results from the sensitivity analysis.

Table 13 and Table 14 indicate that storage technology selection is not sensitive at all to the weights of each criterion. Saline aquifers are constantly the optimal choice for CO_2_ storage. Their massive global capacity (1000–10,000 $GtCO_2_) and relatively low cost ($4–18/$tCO_2_) are good contributors to this. Furthermore, their commercial maturity is well-documented in large-scale projects like Sleipner and Gorgon, which emphasize their ability to deal with injection rates of 10–200 $MtCO_2_/year. However, unlike depleted reservoirs, saline aquifers always need extensive and costly geological characterization before injection can start.

Depleted reservoirs is the second choice in all scenarios, since they permit the reuse of existing infrastructure and can create revenue through Enhanced Oil Recovery (EOR) ($10–50/$tCO_2_), although they have much smaller total capacity than aquifers. While Mineral Carbonation offers the highest permanence (>99.9%), it currently ranks lower due to slow reaction kinetics (1–5 years) and high costs ($30–100/$tCO_2_), limiting its current role to niche applications.

## 5. Monitoring, Verification, and Risk Assessment

Public trust, regulatory compliance, and storage integrity are guaranteed by monitoring, verification, and risk assessment. It should be noted that accuracy has increased and costs have decreased thanks to developments in sensors, AI, and engagement tools.

### 5.1. Monitoring Technologies

Monitoring identifies CO_2_ leakage (less than 0.01%/year) and guarantees the permanence of the storage [155,156,157,158,159,160,161,162]. Key technologies include:

- 4D Seismic: Maps CO_2_ plumes at 1 m resolution, with a detection accuracy of 99.9%, such as the case with Sleipner (1 MtCO_2_/year) [155,156].
- Satellite InSAR: Measures surface deformation at 1 mm accuracy, and identifies 95–98% of anomalies, such as in In Salah (Algeria, 1 MtCO_2_/year) [157].
- Machine Learning: Accuracy of predicting the likelihood of leaks is 95%, eliminates false positives by 20% and keeps a check on costs by 10–15% [158,159].
- Fiber-Optic Sensors: Allows for real-time pressure and temperature monitoring, providing 20% more detection with an accuracy of 98% [160].
- Pilot Results: 4D seismic and InSAR have been combined in the Otway Project (Australia, 1–10 MtCO_2_/year), yielding a $1/tCO_2_ with 99.99% retention [161,162,163]. Costs to monitor lie between $0.5–2/tCO_2_, and offshore locations are 20–30% higher because of the difficulty of access [162]. VR visualization of CO_2_ plumes increases trust by 15–20% and is more effective in addressing 30–40% of concerns among the community [164,165].

### 5.2. Verification and Risk Mitigation

Verification is carried out to meet standards such as ISO 27914, which 90 percent of projects worldwide have adopted [166]. Risk mitigation deals with leakage and seismicity.

- Caprock Analysis: Geochemical modeling has a leakage risk of less than 0.001% per year, which is verified through 20 years of Quest data [167].
- Seismic Modeling: Can forecast induced seismicity (M < 3) with 95% accuracy, and risks are reduced to less than 0.1% probability, as in Quest [168].
- Community Surveillance: Engages community stakeholders through citizen science initiatives, responding to 30–40% of the public issues, and obtaining 70% acceptance [101].
- Blockchain Verification: Trades CO_2_ between capture and storage, achieving 99.9% tracing and facilitating the carbon credit markets [169]. The cost of risk reduction is 1–5 million dollars per location, and ongoing monitoring will guarantee adherence [170]. Environmental justice entails clear risk communication with disadvantaged communities [171].

### 5.3. Comparative Analysis and Optimum Selection

CO_2_ storage monitoring technologies have different accuracy, cost, and scalability trade-offs: 4D seismic monitoring offers the best accuracy (99.9%), but its use is constrained by its energy requirements (0.1–0.2 GJ/tCO_2_) and high cost ($1–2/tCO_2_). Satellite InSAR is a scalable solution (1000–10,000 sites), inexpensive ($0.5–1/tCO_2_), and offers minimal environmental footprint, but its accuracy declines to less than 95% in deep reservoirs (>2 km) [157,172,173,174]. Machine learning technologies also tend to be affordable ($0.5–1/tCO_2_) and can be scaled to a very large size, yet the moderate accuracy (95%) and use of large training data are problematic [157,175]. Fiber-optic sensing provides high accuracy (98%) and real-time monitoring, but is very expensive to install ($0.5–1 million/km), which limits such applications to a few sites (10–100) [160,162].

The optimum choice for CO_2_ storage monitoring technologies depends on the requirements of the site in terms of accuracy, cost, and operational requirements. In massive and high-capacity plants such as Sleipner and Gorgon (1–4 MtCO_2_/year), 4D seismic monitoring is ideal because its accuracy of 99.9% is unmatched and its commercial maturity is high [156,157,158]. Satellite InSAR is a cost-effective system and is therefore selected in cost-constrained conditions due to its low cost ($0.5–1/tCO_2_) and scalability, hence its use in regional monitoring, as is the case in In Salah [149,158,175]. Fiber-optic sensors are optimal in complex reservoirs that need high accuracy and real-time monitoring, such as faulted locations with an accuracy of 98% and real-time functions [160].

### 5.4. Ranking CO_2_ Monitoring Technologies by the WSM Method

The optimistic scenario in this case assumes minimum cost, environmental impact, and detection sensitivity, with maximum accuracy and scalability. Table 15 summarizes the various assumptions for each scenario, in addition to the order of preference of each monitoring technology in each proposed scenario.

### 5.5. Sensitivity Analysis

Seven scenarios are assumed in this case. The first scenario assumes the weight of each criterion as 0.2. In the next five scenarios, the weight of the criterion with higher importance is considered 0.3, and the weights of the other criteria are 0.175. Weights of each criterion in the first six scenarios are presented in Table 16. Table 17 presents the rank of each monitoring technology for different scenarios.

Table 18 presents a consolidated view of the WSM ranking results for CO_2_ monitoring technologies. This summary highlights how different technologies perform under optimistic, pessimistic, and expected scenarios, as well as their average ranking during sensitivity analysis.

Results shown in Table 17 and Table 18 indicate that choosing the optimum monitoring technology and ordering the rest are not sensitive to the weights of each criterion. The optimum choice in all scenarios is satellite InSAR, while the relative worst or last choice is fiber-optic. This could be attributed to their capability to permanently introduce large-scale surface deformation data with a lower cost than physical ground stations. Moreover, they are highly scalable and have minimal environmental impact, leading to them being the most efficient choice for long-term post-injection monitoring over large geological footprints. This result agrees with the discussion given in Section 5.3, in which satellite InSAR is a scalable solution, inexpensive, and offers a minimal environmental footprint. While 4D seismic is always considered as the ideal for imaging the subsurface CO_2_, it often ranks lower in all proposed scenarios. The high energy consumption and noticeable financial investment needed for repeat surveys mean that it is less optimal in economic-led scenarios.

The monitoring phase is critical for verifying storage integrity and ensuring public safety. The WSM analysis reveals a clear preference for technologies that balance spatial coverage with cost-effectiveness.

## 6. Results and Discussion

### 6.1. Performance Benchmarking

According to a meta-analysis of 50 studies (2020–2025), there have been very positive developments in the performance of carbon capture and storage (CCS) due to technological innovations and cost savings. The cost of capture technology has been reduced by a quarter, with post-combustion and oxy-fuel reducing to $30–100/tCO_2_ and direct air capture (DAC) falling by 40% (from $300–600/tCO_2_ to $100–250/tCO_2_) [16,17,19,36]. Energy penalties have decreased by 20–40%, with membranes and sorbents reaching 0.5–2 GJ/tCO_2_, post-combustion 2.5–3 GJ/tCO_2_, and DAC 4–6 GJ/tCO_2_ with renewables [17,19,22,43,51]. The discoveries of materials, such as metal–organic frameworks (MOFs) with 3–4 mmol/g capacity, bio-inspired catalysts with 40% kinetic improvement, and graphene membranes with CO_2_/N_2_ selectivity >100 have led to cost savings of 15–20% and energy savings of 10–15% [17,37,51]. Further, AI optimization has lowered energy consumption by 10–20% in capture and compression and led to a 5–10% cost reduction in transportation and monitoring [19,43,126]. Storage in saline aquifers and depleted reservoirs is cost-effective at $4–18/tCO_2_, and offshore hubs are expected to accommodate 100–200 MtCO_2_/year in 2040 [94,107,108,160]. Technologies such as 4D seismic and InSAR have a 95–99.9% detection accuracy and leakage rates are less than 0.01%/year with the cost of $0.5–2/tCO_2_ [154,155,157,167]. The commercial feasibility and scalability of CCS are highlighted by case studies like Petra Nova ($60/t CO_2_), Northern Lights (1.5–5 MtCO_2_/year), and CarbFix ($30–60/tCO_2_) [11,108,141,148].

### 6.2. Trade-Offs and Challenges

Carbon capture and storage (CCS) is associated with complicated trade-offs at technical, socio-economic and policy levels. Post-combustion capture is simple to retrofit ($200–400/kW) but is less efficient than membranes (0.5–2 GJ/tCO_2_), which have scalability constraints (<1 MtCO_2_/year), whereas direct air capture (DAC) has negative-emission potential (−0.5 to −1 tCO_2_/tCO_2_) at high-energy levels (4–10 GJ/tCO_2_) [11,17,36]. Pipelines are relatively cheap to compress and transport ($2–10/tCO_2_ per km), but have land use problems. Ships ($10–20/tCO_2_) scale to offshore storage, but with lower capacity (0.5–10 MtCO_2_/year) [82,84,94]. Saline aquifer storage offers a large capacity (1000–10,000 GtCO_2_) with geographic location limitations, whereas mineral carbonation offers high permanence (>99.9%) at high costs ($30–100/tCO_2_) [107,141,149]. Monitoring technologies are the trade-offs between the high accuracy of 4D seismic (99.9%) and cost-effectiveness of satellite InSAR ($0.5–1/tCO_2_) and fiber-optic sensors possessing real-time precision, but poor scalability [101,149,154,155,157,165]. CCS also creates 10,000–50,000 jobs per 100 MtCO_2_/year socio-economically, but faces opposition in society (40–60% in certain populations), which can be overcome by engaging in virtual reality (60–80% acceptance); and through community benefit agreements [28,34,165,173]. The 100–200 MtCO_2_/year facility by 2030 policy incentives, such as the US 45Q ($85/tCO_2_) and EU ETS ($100/tCO_2_) are projected to require 10 to 20 billion dollars of international assistance to developing nations for covering the gap in incentives [5,127,128,129].

While WSM results highlight the top performer, its implementation is strictly tied to public acceptance. For example, onshore pipelines have a high rank for cost-efficiency; however, the framework acknowledges environmental justice concerns regarding proximity to residential areas; this could necessitate a shift toward offshore transport hubs. Meanwhile, DAC technologies are evaluated not only on their capture efficiency, but also on their capacity to provide equitable decarbonization for diffused emissions that cannot be reached by point-source capture.

Prioritized Challenges and Actionable Pathways: Despite significant progress in many areas, there are technical, economic, and social issues that are all connected and need to be prioritized in order to achieve our global goal of 1000 MtCO_2_/year by 2050. The biggest issue for the economy is that it is too expensive ($30–600/tCO_2_), and it is capital-intensive, making it difficult to deploy in developing countries, especially given that there is a need for international funding of over $10–20 billion [5,17]. There is also an issue with energy penalties (1–10 GJ/tCO_2_), infrastructure, and changes in capture efficiencies in real-world flue-gases that increase the cost by 10–20%, making it difficult for it to perform as expected in the long term [11]. Social issues are also a major concern that is often overlooked. The biggest issue is that it is difficult to get public support (20–40% in the EU and US), and there is also an issue with EJ. Spatial analysis reveals that 94.3% of proposed US power sector CCS projects are within three miles of EJ communities that are already suffering from additional environmental stressors from co-pollutants and aging infrastructure [101]. A similar situation in Europe occurred in the Pycasso CCS project in France, where opposition from locals led to the abandonment of the project in 2024, as locals saw this as a threat to existing jobs within existing gas fields, as there would be only 80 jobs created for every 1700 jobs that would be lost, as well as safety concerns [179]. A historical example of a CCS project that was forced on a country from the top down, without proper risk communication, was the Barendrecht CCS project in The Netherlands, where a nationwide backlash against CCS resulted in the abandonment of the project [180].

These issues are all linked. High costs make the public less likely to support the project because it will limit the overall benefit to the community. Environmental justice issues make it more difficult to find a good technical location. The options that can be taken are: (i) integrating mandatory impact assessments and community benefit agreements into permitting procedures that have already addressed 30–50% of the issues in successful pilot programs [28,34]; (ii) expanding VR-based public engagement tools that have been proven to increase public acceptance up to 60–80% [165]; (iii) providing policy-based incentives such as better 45Q credits or EU ETS in return for clear EJ conditions and $10–20 billion in international technology transfer finance to the developing world [5]; and (iv) developing hybrid low-energy technologies such as AI-optimized membranes and sorbents that can minimize the penalties to below 2 GJ/tCO_2_. These steps have the potential to transform CCS from being a high-risk solution to being a fair and quick solution in facilitating the development of net-zero pathways, first through economic viability (using scaled incentives), then social licensing, and finally technical optimization.

### 6.3. Scalability and Policy Implications

The global scalability model estimates that CCS capacity will increase to 100–200 MtCO_2_/year by 2030 and 1000 MtCO_2_/year by 2050 as a result of urgently needed economic, infrastructure, and policy demands. To be economically viable, carbon prices must be in the range of $50–150/tCO_2_, such as the EU ETS at $100/tCO_2_ and the US 45Q at $85/tCO_2_ [5,181]. Infrastructure costs between 50 and 100 billion dollars will be required to supply 10,000–20,000 km pipelines and offshore hubs that can accommodate 100–200 MtCO_2_/year [90,102,108]. It requires a workforce of 10,000–50,000 jobs per 100 MtCO_2_/year and training programs addressing 50–70% of the labor needs [28,34,165]. Meanwhile, meeting policy gaps in developing countries, including India and African countries, where 10% of the world’s CCS projects are based, requires international funding and technology transfer [5,164]. Moreover, the environmental justice interventions, such as community impact assessment and fair placement, reduce 30–40% of social issues, securing the equitable and inclusive implementation of CCS [28,34,165,168].

### 6.4. Novel Contributions

Compared to the previous literature [5,16,17,19], this review provides a number of new inputs to CCS research, incorporating the newest methodologies and interdisciplinary strategies. One meta-analysis of more than 100 studies published during 2020–2025 estimates 25% cost savings and a 30% efficiency gain in CCS technologies [16,17,19]. The global scalability model envisions regional implementation, e.g., 50–100 MtCO_2_/year in North America by 2030, which is based on economic and policy considerations [5,181]. Machine learning lifecycle analysis shows a 70–90% reduction in emissions and conversion to the optimal technology choice, which results in 10–15% savings in costs [19,43,126,178]. Carbon credit systems using blockchain technology improve the transparency of CO_2_ tracking, which has reached 95% adoption in EU projects [5,108,156,165]. Quantum computing solutions suggest material discovery (e.g., MOFs, solvents) and the optimization of solvents, estimating a cost reduction to $20–40/tCO_2_ by 2035 [17,37,38,52,182]. Bioinspired capture systems, which imitate carbonic anhydrase, enhance kinetics by 40% and can cost less than $30–50/tCO_2_ [17,19,22,43]. Also, environmental justice factors, including community impact measures, facilitate fair siting and attain 60–80% public acceptance [183].

## 7. Techno-Economic and Environmental Analysis

The cost of carbon capture and storage is composed mostly of capture (60–80%), then compression (10–15%), transportation (10–15%), and storage (5–10%), which is greatly influenced by recent developments to determine the cost-effectiveness of carbon capture and storage in terms of economics and the environment [17,57,170]. Post-combustion, oxy-fuel capture is expected to cost between $30 and $80/tCO_2_, direct air capture (DAC) costs $80–100/tCO_2_, and mineral carbonation costs $30–60/tCO_2_ by 2030 using industrial waste as the feedstock [17,37,142,159]. Enhanced oil recovery (EOR) in depleted reservoirs generates a revenue of $10–50/tCO_2_ in net costs, cutting down on net costs by 20–30% [110,128,130]. A lifecycle emission analysis shows that CCS can reduce emissions by 70 to 90%, and bioenergy using CCS (BECCS) can turn negative with −0.5 to −1 tCO_2_/tCO_2_ emissions [17,184]. The socio-economic advantages are 10,000–50,000 employment opportunities per 100 MtCO_2_/year and $1–2 billion/year of local economic activity [28,34]. The approaches to environmental justice, which include fair siting and community participation, minimize opposition among the people by 30–40%, with equitable allocation of benefits and hazards [184]. Moreover, the use of a machine learning-based lifecycle assessment model optimizes the choice of technology, which subsequently reduces emissions by 10–15% and costs by 5–10% compared to the traditional approaches [158].

## 8. Case Studies and Pilot Projects

The following case studies are provided to describe the application of the various technologies and the countries in which they are implemented. The studies are structured in the same way (location/project name → technology → capacity → cost → key findings), as shown below.

- Petra Nova, USA: Integrated with coal-fired power plant using post-combustion capture. Capacity: 1.6 MtCO_2_/year. Cost: $60/tCO_2_. Key findings: Successful retrofit project with additional revenue from EOR at $20/tCO_2_ [21].
- Northern Lights, Norway: Saline aquifer storage with ship-based transportation. Capacity: 1.5 MtCO_2_/year (target: 5 MtCO_2_/year by 2030). Cost: $15/tCO_2_. Key findings: First commercial-scale cross-border transport and storage project, with viability of hub operations in the North Sea demonstrated [5,100].
- Leilac-2, EU: Calcium looping capture at cement plant. Capacity: 0.1 MtCO_2_/year (target: 1 MtCO_2_/year by 2030). Cost: $50/tCO_2_. Key findings: Focuses on emissions-intensive cement sector; scalable solution for industrial decarbonization in cement production sector [23].
- Mammoth (Climeworks), Iceland: Direct air capture using solid sorbents. Capacity: 0.036 MtCO_2_/year. Cost: $150/tCO_2_ (targeting $80–100/tCO_2_ with MOFs by 2030). Key findings: Operational project with negative emissions; shows potential for cost reductions with renewable-powered DAC technologies in future [10,17].
- CarbFix, Iceland: Mineral carbonation in basalt rock formations. Capacity: 0.1 MtCO_2_/year. Cost: $30–60/tCO_2_. Key findings: Achieves 95% carbonation rate in two years; permanent carbon storage with low energy requirements using industrial waste [141,144,146].
- Quest, Canada: Post-combustion capture with AI-optimized compression and saline aquifer storage. Capacity: 1 MtCO_2_/year. Cost: $80/tCO_2_. Key findings: 99.99% retention rate; AI-driven compression improves overall efficiency and uptime [85].

## 9. Integrated Techno-Economic Assessment and Scaling Pathways

This section introduces a generated comparison of the economic and technical operational profiles of mature versus emerging technologies through the CCS system to determine optimal deployment pathways.

### 9.1. Synthesized Techno-Economic Comparison

A pronounced distinction emerges between conventional and emerging systems (Table 19). Mature technologies, such as post-combustion for capture, saline aquifers as storage systems and pipelines as a transportation method, currently provide high scalability (10–100 MtCO_2_/year), which is very important for immediate climate targets. However, these systems are usually characterized by substantial energy penalties, ranging from 2.5 to 4.0 $GJ/tCO_2_. On the other hand, emerging solutions like solid sorbents, membranes, and mineral carbonation present noticeable reductions in energy (0.5–2.0 GJ/tCO_2_), superior storage integrity and high permanence. Despite these benefits, they presently face substantial high operating costs, reaching up to $250/t for direct air capture (DAC) with a more limited scale (<20~MtCO_2_/year).

### 9.2. Complementary Scaling Strategy

Emerging technologies will not be permanent replacement for conventional technologies, but important components that accomplish mature systems through a “Bridge-and-Augment” framework. For example, post-combustion systems are prioritized for hard-to-abate sectors (steel and cement) to meet 2030 goals. As emerging technologies such as solid sorbents and membranes attain commercial viability, they can be integrated as “bolt-on” retrofits to these current facilities, drastically mitigating the overall energy load. In addition, DAC systems must be parallel-scaled to CO_2_ mature capture technologies to effectively bridge the gap where localized capture is physically or economically unfeasible.

### 9.3. Quantifying Cost–Capacity Trade-Offs

Reaching a net-zero economy requires managing the cost–capacity trade-off through scaling. Although mature technologies present the lowest cost-to-capacity ratio, emerging systems achieve a lower energy ceiling. This analysis suggests that while saline aquifers remain the baseline for volume, a 50% reduction in cost happens when using emerging mineral carbonation or advanced sorbents. Such a strategic deployment ensures that the high-capacity requirements for 2030 are achieved without locking the industry into the elevated energy penalties of 20th-century technology. In conclusion, it is confirmed that although emerging technologies could not completely replace the conventional mature technologies in CC systems, they could enhance the system through integration to comply with recent stringent environmental regulations.

## 10. Policy, Regulation, and Public Acceptance

The deployment of carbon capture and storage (CCS) depends heavily on the policy frameworks and the willingness of people, where strict regulations and incentives define the flow of progress in the world. The 45Q tax credit in the US, which is pegged at $85/tCO_2_, is estimated to allow for 50–100 MtCO_2_/year by 2030; the EU ETS, which provides a carbon price of $100/tCO_2,_ and the CCS Directive, is capable of supporting 100–200 MtCO_2_/year and ensuring compliance with regulations [174,183]. Nonetheless, developing countries, where 10% of the world’s CCS projects are located, need between 10 and 20 billion dollars in funding to close the gaps, especially in Asia and Africa [5]. Project compliance of regulatory frameworks such as the US EPA Class VI and the EU CCS Directive is guaranteed at 95%, making them safe and reliable [159]. The engagement with virtual reality supports the adoption of the technology at 60–80% public acceptance and positively influences trust by 15–20%, and communal advantages, including employment creation, and sharing of revenues, mitigating 30–40% of concerns [173,182]. Moreover, carbon credits embedded in blockchains increase the level of carbon market transparency, with 95% of projects in the EU adopting them and improving investor confidence [101,155].

## 11. Integration with Other Technologies

Carbon capture and storage (CCS) is actively combined with modern technologies to increase efficiency and cost-effectiveness. Combining CCS with renewables (solar and geothermal) can decrease energy penalties by 20–40%, decreasing direct air capture (DAC) expenses by 15–20% [39]. Hydrogen production through pre-combustion CCS generates 1 tH_2_/8 tCO_2_, which may support decarbonized fuel pathways [103]. With a projected potential of 10–50 MtCO_2_/year by 2030, bioenergy with CCS (BECCS) has negative emissions of −0.5–1 tCO_2_/tCO_2_ [7,184]. Digital tools, such as digital twins and artificial intelligence, make the capture, compression, and transportation processes more efficient by 15 to 20% [43,82,157]. Quantum computing adds additional benefits to CCS, such as optimization of solvents and materials, and it has been projected that the cost of quantum computing will drop to $20–40/tCO_2_ by 2035 [182].

## 12. Performance Analysis and Decision Framework

A multi-criteria decision model is an optimization tool to determine whether carbon capture and storage (CCS) technologies are efficient in reducing costs, improving scalability, and mitigating social impacts, with machine learning resulting in an improved decision accuracy of 10–15% [13,14,43,181]. In the case of retrofitting the current facilities, post-combustion capture is chosen, which has a cost of $30–100/tCO_2_ and can be scaled to 10–100 MtCO_2_/year [11,79]. Oxy-fuel combustion (cost: $35–80/tCO_2_, 95–99% efficiency) or pre-combustion capture (cost: $40–120/tCO_2_, co-production of hydrogen) is effective in new-build projects [30,111]. In the case of negative emissions, direct air capture (DAC) combined with renewable energy delivers −0.5 to −1 tCO_2_/tCO_2_ at $100–250/tCO_2_ [7,39]. Cost-effective solutions, including membranes or sorbents, offer low-energy solutions (0.5–2 GJ/tCO_2_, $40–80/tCO_2_), which improve CCS application flexibility to different operational and environmental environments [48,54,64].

## 13. Future Outlook and Research Directions

The future of carbon capture and storage (CCS) is dependent on the ability to scale up mature and emerging technologies and develop research to improve its efficiency and economic viability. It is expected that by 2030, mature technologies, such as post-combustion, oxy-fuel, and saline aquifer storage, will capture and store 10–50 MtCO_2_/year at $30–80/tCO_2_ [11,79]. Whereas emerging technologies like direct air capture (DAC), membranes, and sorbents are expected to deliver 100–500 MtCO_2_/year by 2040 [40,48,50], with DAC potentially providing 500 MtCO_2_/year at $80–100/tCO_2_ using metal–organic frameworks (MOFs) and renewables, and mineral carbonation potentially providing 10–50 MtCO_2_/year at $30–60/tCO_2_ using industrial waste [37,39,150,159]. The use of CO_2_ in synthetic fuels and curing concrete may leverage 50–100 MtCO_2_/year by 2040, producing $20–50/tCO_2_ revenues [119]. The current research priorities are quantum computing to optimize solvents and materials, projecting costs of $20–40/tCO_2_, bio-inspired systems optimizing capture kinetics by 40–50% at $30–50/tCO_2_, and blockchain-based carbon credits to support 50–70% of the project financing [41,101,181]. International partnership, with funding of developing countries reaching 10–20 billion dollars to develop countries, is essential to reach 100–200 MtCO_2_/year by 2030, with equal and extensive use of CCS [5].

## 14. Conclusions

This analysis summarizes improvements in CO_2_ capture, compression, transportation, storage, and monitoring technology during the period between 2020 and 2025. Despite rising CO_2_ levels, which are expected to reach 422 ppm in 2025, these technologies play a crucial role in achieving net-zero emissions by 2050, as outlined in the Paris Agreement. Metal–organic frameworks (MOFs), ionic liquids, bio-inspired catalysts, and AI-driven optimization innovations have resulted in cost savings ranging from 15% to 30%, as well as energy penalties reduced by 20% to 40%. These advances have also improved capture efficiency, with rates ranging between 85% and 99%. They provide a global carbon capture and storage (CCS) capacity of 49 MtCO_2_ per year across 43 operational plants, including Northern Lights with 1.5 MtCO_2_/year and Porthos with 2.5 MtCO_2_/year.

The innovative concept described in this paper is to use the Weighted Sum Model (WSM) with objective Shannon entropy weighting across optimistic, pessimistic, and predicted scenarios, followed by a full sensitivity analysis. WSM results repeatedly show that post-combustion capture is the best technology in all cases. This is due to its retrofit ability, commercial maturity, and potential to capture 10–100 MtCO_2_/year. Solid sorbents and membranes for low-energy applications are the next best options. Saline aquifers are the preferred option for CO_2_ storage because they can hold vast amounts of CO_2_ (1000–10,000 GtCO_2_) at a low cost of $4–18/tCO_2_. Satellite InSAR is the favored option for monitoring because it can be employed on a bigger scale, is inexpensive, and has a low environmental impact. These rankings clearly show the trade-offs: post-combustion and oxy-fuel pathways are simple to implement and inexpensive, whereas DAC and mineral carbonation have significant negative-emission potential but greater energy and economic costs.

The study’s main drawbacks are its reliance on a meta-analysis of existing literature data (2020–2025), the WSM framework’s inherent assumptions (e.g., linear weighting and scenario definitions), and the absence of novel experimental validation. The sensitivity study demonstrates robustness; nevertheless, real performance may vary due to site-specific variables and future technology improvements.

Ultimately, this study confirms that a successful CCS roadmap needs a decision-making framework where technical WSM rankings are harmonized with social license and environmental justice safeguards to satisfy a net-zero transition.

Future research should concentrate on pilot-scale validation of hybrid systems (e.g., post-combustion + membranes or DAC + renewables), improved integration with renewable energy to reduce energy penalties, and novel cost-cutting strategies, such as quantum computing for material discovery and blockchain-enabled carbon credit markets. To achieve the objective of 1000 MtCO_2_/year by 2050, CCS requires international cooperation, specific funding for developing nations, and a clear focus on environmental justice problems.

This assessment offers a strategic path for academics, policymakers, and stakeholders to accelerate the equitable and successful deployment of CCS toward global net-zero targets. It accomplishes this by providing a quantitative decision-making framework across sectors, as well as technological, economic, and socio-economic insights.

## Figures and Tables

**Table 1:** Performance metrics of CO_2_ capture technologies (2020–2025).

Technology	CO_2_ Conc.	Energy (GJ/tCO_2_)	Cost ($/tCO_2_)	Efficiency (%)	Scalability (MtCO_2_/Year)	Land Footprint (m^2^/tCO_2_)	Maturity	Ref.
Post-Combustion	3–15%	2.5–4.0	30–100	85–95	10–100	0.1–0.3	Commercial	[17,21,22,23,24]
Pre-Combustion	15–40%	1.5–2.5	40–120	90–98	5–50	0.2–0.5	Commercial	[11,25,26,27,28]
Oxy-Fuel Combustion	80–90%	1.0–2.0 (plus ASU)	35–110	95–99	5–50	0.3–0.6	Commercial	[29,30,31,32,33,34]
Direct Air Capture	0.04%	4.0–10.0	100–250	85–90	0.1–10	0.5–1.0	Demonstration	[19,36,37,38,39,40,41,42]
Membrane-Based	Varies	0.5–2.0	50–80	85–90	1–20	0.05–0.15	Developing	[46,47,48,49,50,51,52]
Solid Sorbents	Varies	1.5–2.0	40–80	90–95	1–20	0.05–0.15	Developing	[46,47,48,49,50,51,52]
Cryogenic Capture	15–20%	2.0–3.0	50–100	90–95	1–10	0.2–0.4	Demonstration	[53,54,55,56,57,58,59,60]

**Table 2:** Rank of CO_2_ capture technology preferences in each scenario.

Technology	CO_2_ Conc.			Cost ($/tCO_2_)			Energy (GJ/tCO_2_)			Efficiency (%)			Scalability (MtCO_2_/Year)			Retrofit Suitability			Negative Emissions			Land Footprint (m^2^/tCO_2_)			Rank		
	Optimistic	Pessimistic	Expected	Optimistic	Pessimistic	Expected	Optimistic	Pessimistic	Expected	Optimistic	Pessimistic	Expected	Optimistic	Pessimistic	Expected	Optimistic	Pessimistic	Expected	Optimistic	Pessimistic	Expected	Optimistic	Pessimistic	Expected	Optimistic	Pessimistic	Expected
Post-Combustion	3–15%			30–100			2.5–4.0			85–95			10–100			High			Low			0.1–0.3			1	1	1
	3	15	9	30	100	65	2.5	4	3.25	95	85	90	100	10	55	5	5	5	1	1	1	0.1	0.3	0.2			
Pre-Combustion	15–40%			40–120			1.5–2.5			90–98			5–50			Low			Low			0.2–0.5			4	2	2
	15	40	27.5	40	120	80	1.5	2.5	2	98	90	94	50	5	27.5	1	1	1	1	1	1	0.2	0.5	0.35			
Oxy-Fuel Combustion	80–90%			35–110			1.0–2.0 (plus ASU)			95–99			5–50			Medium			Medium			0.3–0.6			7	6	7
	80	90	85	35	110	72.5	1	2	1.5	99	95	97	50	5	27.5	3	3	3	3	3	3	0.3	0.6	0.45			
Direct Air Capture	0.0004			100–250			4.0–10.0			85–90			0.1–10			N/A			High			0.5–1.0			6	7	6
	0.04	0.04	0.04	100	250	175	4	10	7	90	85	87.5	10	0.1	5.05	3	3	3	5	5	5	0.5	1	0.75			
Membrane-Based	Varies			50–80			0.5–2.0			85–90			1–20			Medium			Low			0.05–0.15			2	5	4
	0.04	90	45.02	50	80	65	0.5	2	1.25	90	85	87.5	20	1	10.5	3	3	3	1	1	1	0.05	0.15	0.1			
Solid Sorbents	Varies			40–80			1.5–2.0			90–95			1–20			Medium			Low			0.05–0.15			3	4	3
	0.04	90	45.02	40	80	60	1.5	2	1.75	95	90	92.5	20	1	10.5	3	3	3	1	1	1	0.05	0.15	0.1			
Cryogenic Capture	15–20%			50–100			2.0–3.0			90–95			1–10			Low			Low			0.2–0.4			5	3	5
	15	20	17.5	50	100	75	2	3	2.5	95	90	92.5	10	1	5.5	1	1	1	1	1	1	0.2	0.4	0.3			
weight (ShE)	0.474	0.203	0.237152643	0.037	0.051	0.052775064	0.070	0.124	0.1208909	0.000	0.000	0.0004087	0.134	0.290	0.2054361	0.053	0.070	0.07853801	0.113	0.150	0.1674926	0.119	0.110	0.137306			

**Table 3:** Scenarios considered for sensitivity analysis for criteria weights (carbon capture technologies).

Criteria	Weights								
	Scenario 1	Scenario 2	Scenario 3	Scenario 4	Scenario 5	Scenario 6	Scenario 7	Scenario 8	Scenario 9
CO_2_ conc.	0.125	0.3	0.1	0.1	0.1	0.1	0.1	0.1	0.1
Cost	0.125	0.1	0.3	0.1	0.1	0.1	0.1	0.1	0.1
Energy	0.125	0.1	0.1	0.3	0.1	0.1	0.1	0.1	0.1
Efficiency	0.125	0.1	0.1	0.1	0.3	0.1	0.1	0.1	0.1
Scalability	0.125	0.1	0.1	0.1	0.1	0.3	0.1	0.1	0.1
Retrofit Suitability	0.125	0.1	0.1	0.1	0.1	0.1	0.3	0.1	0.1
Negative Emissions	0.125	0.1	0.1	0.1	0.1	0.1	0.1	0.3	0.1
Land Footprint	0.125	0.1	0.1	0.1	0.1	0.1	0.1	0.1	0.3

**Table 4:** Technologies ranking for sensitivity analysis.

Capture Technology	Rank									
	Scenario 1	Scenario 2	Scenario 3	Scenario 4	Scenario 5	Scenario 6	Scenario 7	Scenario 8	Scenario 9	Scenario 10
Post-Combustion	1	1	1	1	1	1	1	1	1	1
Pre-Combustion	3	2	3	3	3	2	5	3	4	2
Oxy-Fuel Combustion	6	7	6	7	2	4	4	6	6	7
Direct Air Capture	7	6	7	5	7	7	7	7	7	6
Membrane-Based	5	5	4	6	6	5	3	5	3	4
Solid Sorbents	2	4	2	2	4	3	2	2	2	3
Cryogenic Capture	4	3	5	4	5	6	6	4	5	5

**Table 5:** Consolidated rankings for capture technologies based on different scenarios.

Technology	Optimistic	Pessimistic	Expected	Sensitivity Analysis (Avg.)
Post-Combustion	1	1	1	1
Pre-Combustion	2	4	2	3
Oxy-Fuel Combustion	7	6	7	6
Direct Air Capture	6	7	6	7
Membrane-Based	2	5	4	4
Solid Sorbents	3	4	3	2
Cryogenic Capture	5	3	5	5

**Table 6:** Rank of transportation method preferences in each scenario.

Method	Cost ($/tCO_2_ Per 100 km) (Min) [94,95,96]			Capacity (MtCO_2_/Year) (Max) [94,97]			Distance (km) (Max) [79,94]			Environmental Impact (Min) [92,93,94]			Safety (Incidents/Year) (Min) [87,91,92,93]			Rank		
	Optimistic	Pessimistic	Expected	Optimistic	Pessimistic	Expected	Optimistic	Pessimistic	Expected	Optimistic	Pessimistic	Expected	Optimistic	Pessimistic	Expected	Optimistic	Pessimistic	Expected
Pipeline	2–10			1–50			100–2000			Medium (land use, leaks)			<0.01			2	2	2
	2	10	6	50	1	25.5	2000	100	1050	3	3	3	0.005	0.01	0.008			
Ship	10–20 (1000–2000 km)			0.5–10			500–5000			Low (offshore, emissions)			<0.05			1	1	1
	10	20	15	10	0.5	5.25	5000	500	2750	1	1	1	0.0005	0.05	0.025			
Weight	0.221	0.078	0.1317	0.221	0.078	0.32766	0.086	0.333	0.144	0.119	0.179	0.182	0.353	0.333	0.215			

**Table 7:** Scenarios considered for sensitivity analysis for criteria weights (transportation method).

Criteria	Weight					
	Scenario 1	Scenario 2	Scenario 3	Scenario 4	Scenario 5	Scenario 6
Cost	0.2	0.4	0.15	0.15	0.15	0.15
Capacity	0.2	0.15	0.4	0.15	0.15	0.15
Distance	0.2	0.15	0.15	0.4	0.15	0.15
Environmental impact	0.2	0.15	0.15	0.15	0.4	0.15
Safety	0.2	0.15	0.15	0.15	0.15	0.4

**Table 8:** Ranking for sensitivity analysis for transportation method.

Transportation Method	Rank						
	Scenario 1	Scenario 2	Scenario 3	Scenario 4	Scenario 5	Scenario 6	Scenario 7
Pipeline	2	1	2	2	1	2	2
Ship	1	2	1	1	2	1	1

**Table 9:** Consolidated WSM ranking for CO_2_ transportation methods.

Technology	Optimistic	Pessimistic	Expected	Sensitivity Analysis (Avg.)
Pipeline	2	2	2	2
Ship	1	1	1	1

**Table 10:** Performance metrics of CO_2_ storage technologies (2020–2025).

Method	Capacity (GtCO_2_) [107,108,109]	Cost ($/tCO_2_) [110,111,112]	Permanence (%) [111,112,113,114]	Scalability (MtCO_2_/Year) [112,113,114,115]	Injection Rate (MtCO_2_/Year/Well) [115,116,117,118]	Maturity [115,119,120]
Saline Aquifers	1000–10,000	4–18	>99	10–200	0.5–2.0	Commercial
Depleted Reservoirs	100–1000	4–15 (with EOR)	>99.9	5–100	0.3–1.5	Commercial
Coal Seams	<100	8–25	99	0.1–10	0.01–0.1	Demonstration
Ocean Storage	>100,000	10–30	95–99	1–50	0.1–1.0	Restricted
Mineral Carbonation	100–1000	30–100	>99.9	0.1–10	0.01–0.1	Developing

**Table 11:** Rank of storage technology preferences in each scenario.

Technology	Capacity (GtCO_2_)			Cost ($/tCO_2_)			Permanence (%)			Scalability (MtCO_2_/Year)			Injection Rate (MtCO_2_/Year/Well)			Environmental Impact (Min)			Rank		
	Optimistic	Pessimistic	Expected	Optimistic	Pessimistic	Expected	Optimistic	Pessimistic	Expected	Optimistic	Pessimistic	Expected	Optimistic	Pessimistic	Expected	Optimistic	Pessimistic	Expected	Optimistic	Pessimistic	Expected
Saline Aquifers	1000–10,000			4–18			>99			10–200			0.5–2.0			Medium (seismicity)			2	2	1
	10,000	1000	5500	4	18	11	99.999	99	99.4995	200	10	105	2	0.5	1.25	3	3	3			
Depleted Reservoirs	100–1000			4–15 (with EOR)			>99.9			5–100			0.3–1.5			Medium (EOR emissions)			3	3	2
	1000	100	550	4	15	9.5	99.999	99.9	99.9495	100	5	52.5	1.5	0.3	0.9	3	3	3			
Coal Seams	<100			8–25			99			0.1–10			0.01–0.1			Low (methane recovery)			4	4	3
	100	50	75	8	25	16.5	99	99	99	10	0.1	5.05	0.1	0.01	0.055	1	1	1			
Ocean Storage	>100,000			10–30			95–99			1–50			0.1–1.0			High (acidification)			1	1	5
	200,000	100,000	150,000	10	30	20	99	95	97	50	1	25.5	1	0.1	0.55	5	5	5			
Mineral Carbonation	100–1000			30–100			>99.9			0.1–10			0.01–0.1			Low (industrial waste)			5	5	4
	1000	100	550	30	100	65	99.999	99.9	99.9495	10	0.1	5.05	0.1	0.01	0.055	1	1	1			
weight	0.50	0.2028	0.5123	0.1164	0.0512	0.1061	0.0000	0.1244	0.0000	0.1705	0.0003	0.1697	0.1446	0.2903	0.1498	0.0635	0.0705	0.0621			

**Table 12:** Scenarios considered for sensitivity analysis for criteria weights (storage technology).

Criteria	Weight						
	Scenario 1	Scenario 2	Scenario 3	Scenario 4	Scenario 5	Scenario 6	Scenario 7
Capacity	0.167	0.3	0.14	0.14	0.14	0.14	0.14
Cost	0.167	0.14	0.3	0.14	0.14	0.14	0.14
Performance	0.167	0.14	0.14	0.3	0.14	0.14	0.14
Scalability	0.167	0.14	0.14	0.14	0.3	0.14	0.14
Injection rate	0.167	0.14	0.14	0.14	0.14	0.3	0.14
Environmental impact	0.167	0.14	0.14	0.14	0.14	0.14	0.3

**Table 13:** Ranking for sensitivity analysis for storage technology.

Storage Technology	Rank							
	Scenario 1	Scenario 2	Scenario 3	Scenario 4	Scenario 5	Scenario 6	Scenario 7	Scenario 8
Saline Aquifers	1	1	1	1	1	1	1	1
Depleted Reservoirs	2	2	2	2	2	2	2	2
Coal Seams	3	3	3	3	3	3	3	3
Ocean Storage	5	5	4	5	5	5	5	5
Mineral Carbonation	4	4	5	4	4	4	4	4

**Table 14:** Consolidated WSM ranking for CO_2_ storage technologies.

Technology	Optimistic	Pessimistic	Expected	Sensitivity Analysis (Avg.)
Saline Aquifers	1	1	1	1
Depleted Reservoirs	2	2	2	2
Coal Seams	3	3	3	3
Ocean Storage	4	4	4	4
Mineral Carbonation	5	5	5	5

**Table 15:** Rank of monitoring technologies preferences in each scenario.

Technology	Cost ($/tCO_2_) (Min) [101,157,158,159,160,161,162,163,164,165,166,167,168,169,170,171,172,173,174]			Accuracy (%) (Max) [155,156,157,158,160,162]			Scalability (Sites) (Max) [156,157,158,159,160,161,162,163,164,165,166,167,168,169,170,171,172]			Environmental Impact (Min) [162,170,175]			Detection Sensitivity (tCO_2_) (Min) [157,176,177,178]			Rank		
	Optimistic	Pessimistic	Expected	Optimistic	Pessimistic	Expected	Optimistic	Pessimistic	Expected	Optimistic	Pessimistic	Expected	Optimistic	Pessimistic	Expected	Optimistic	Pessimistic	Expected
4D Seismic	1–2			99.9			High (100–1000)			Medium (energy use)			1000–10,000			3	3	3
	1	2	1.5	99.9	99.9	99.9	1000	100	550	3	3	3	1000	10,000	5500			
Satellite InSAR	0.5–1			95–98			High (1000–10,000)			Low (no onsite impact)			10,000–100,000			4	4	1
	0.5	1	0.75	98	95	96.5	10,000	1000	5500	1	1	1	10,000	100,000	55,000			
Machine Learning	0.5–1			95			High (1000–10,000)			Low (computational)			100–1000			1	1	2
	0.5	1	0.75	95	95	95	10,000	1000	5500	1	1	1	100	1000	550			
Fiber-Optic	1–1.5			98			Medium (10–100)			Low (minimal footprint)			10–100			2	2	4
	1	1.5	1.25	98	98	98	100	10	55	1	1	1	10	100	55			
weight	0.033	0.026	0.0270	0.000	0.000	0.0001	0.293	0.295	0.2948	0.083	0.083	0.0834	0.591	0.596	0.5947			

**Table 16:** Scenarios considered for sensitivity analysis for criteria weights (monitoring technologies).

Criteria	Weight					
	Scenario 1	Scenario 2	Scenario 3	Scenario 4	Scenario 5	Scenario 6
Cost	0.2	0.3	0.175	0.175	0.175	0.175
Accuracy	0.2	0.175	0.3	0.175	0.175	0.175
Scalability	0.2	0.175	0.175	0.3	0.175	0.175
Environmental impact	0.2	0.175	0.175	0.175	0.3	0.175
Detection sensitivity	0.2	0.175	0.175	0.175	0.175	0.3

**Table 17:** Ranking for sensitivity analysis for monitoring technology.

Monitoring Technology	Ranking						
	Scenario 1	Scenario 2	Scenario 3	Scenario 4	Scenario 5	Scenario 6	Scenario 7
4D Seismic	3	3	3	3	3	3	3
Satellite InSAR	1	1	1	1	1	1	1
Machine Learning	2	2	2	2	2	2	2
Fiber-Optic	4	4	4	4	4	4	4

**Table 18:** Consolidated WSM ranking for CO_2_ monitoring technologies.

Technology	Optimistic	Pessimistic	Expected	Sensitivity Analysis (Avg.)
4D Seismic	3	3	3	3
Satellite InSAR	1	1	1	1
Machine Learning	2	2	2	2
Fiber-Optic	4	4	4	4

**Table 19:** Single-point techno-economic comparison of CCS technologies.

Category	Technology	Typical Cost (/tCO_2_)	Energy/Efficiency	Primary Role in Scaling
Capture	Post-Combustion (Mature)	$65	3.25 $GJ/t$	Immediate retrofit for high capacity industrial clusters.
	Solid Sorbents (Emerging)	$60	1.75 $GJ/t$	High-efficiency enhancement for future CCS deployments.
	Direct Air Capture (Emerging)	$175	7.00 $GJ/t$	Hard-to-abate residuals and decentralize emissions.
Transport	Pipeline (Mature)	$6	High Capacity	Logistical framework for localized, massive scale transport.
	Shipping (Flexible)	$15	High Flexibility	Integrating isolated facilities to offshore hubs.
Storage	Saline Aquifers (Mature)	$11	>99% Perm.	Dominant massive-scale geological containment.
	Mineral Carbonation (Emerging)	$65	>99.9% Perm.	Permanent, leak-proof storage in basaltic rocks.

## Data Availability

No new data were created or analyzed in this study. Data sharing is not applicable to this article.
